# University of Michigan - Dental Department

**Published:** 1889-07

**Authors:** 


### EDUCATIONAL.

### University of Michigan.—Dental Department.

The fourteenth annual commencement of the Dental Department of the University of Michigan was held in the University Hall, Thursday, June 27th, at the time of and in connection with the commencement of all the other departments of the university. The number of matriculates for the session was 112.

The degree of D.D.S., was conferred on the following named persons by the President of the University, Dr. J. B. Angell.

Albert Edward Anderson.

Robert Burns Avery.

Harry Fielden Briggs.

Frank Seldon Buckley, A.B.

Charles Sumner Buttolph.

George Benton Chester.

George Edward Courtney.

Harry Goodrick Dunaven.

Louis Phillips Hall.

Frank Douglass Harding.

George Byron Hayes, A.B.

Clarence Eugene Henderson.

William Carley Herbert.

George Arthur Holliday.

Horace Nathaniel Holmes.

Edy Randall Johnson.

Jacob William Jungman.

Oscar Calm Kerlin.

Reuben John Kirk.

Charles ShuterMcIndoe.

Edward Cook Mills.

Frank E. Morey.

Charles Franklin Noyes.

Arthur Mowry Potter.

John Scott Rice, M.D., D.D.S.

Arthur Richardson.

Sumner Oliver Sawyer.

Henry Herman Schuhmann.

DeWitt Spalsbury.

Carroll Wesley Staples.

Griffith Pritchard Terry.

Frank Prescott Watson.

Joe Welch.

John H. Williams.

Number Graduating.................................................................. 34.

The total number graduating from all departments was 438.

Dr. George Ross has been appointed Professor of the Practice of Medicine and Dr. Richard L. MacDonnell Professor of Clinical Medicine in McGill University, Montreal.

